# Primary cilia in gastric Gastrointestinal Stromal Tumours (GISTs): an ultrastructural study

**DOI:** 10.1111/jcmm.12067

**Published:** 2013-05-15

**Authors:** Tomás Castiella, Guillermo Muñoz, María José Luesma, Sonia Santander, Mario Soriano, Concepción Junquera

**Affiliations:** a Department of Pathology and Human Histology and Anatomy, Faculty of Medicine, University of ZaragozaZaragoza, Spain; b University Clinic Hospital, IIS AragónZaragoza, Spain; c Department of Electron Microscopy, Príncipe Felipe Research CentreValencia, Spain

**Keywords:** primary cilia, gastric gastrointestinal stromal tumours, ultrastructure

## Abstract

Gastrointestinal stromal tumours (GISTs) are the most common mesenchymal (non-epithelial) neoplasms of the human gastrointestinal (GI) tract. They are thought to derive from interstitial cells of Cajal (ICCs) or an ICC progenitor based on immunophenotypical and ultrastructural similarities. Because ICCs show primary cilium, our hypothesis is based on the possibility that some of these neoplastic cells could also present it. To determine this, an exhaustive ultrastructural study has been developed on four gastric GISTs. Previous studies had demonstrated considerable variability in tumour cells with two dominating phenotypes, spindly and epithelioid. In addition to these two types, we have found another cell type reminiscent of adult ICCs with a voluminous nucleus surrounded by narrow perinuclear cytoplasm with long slender cytoplasmic processes. We have also noted the presence of small undifferentiated cells. In this study, we report for the first time the presence of primary cilia (PCs) in spindle and epithelioid tumour cells, an ultrastructural feature we consider of special interest that has hitherto been ignored in the literature dealing with the ultrastructure of GISTs. We also point out the frequent occurrence of multivesicular bodies (MVBs). The ultrastructural findings described in gastric GISTs in this study appear to be relevant considering the critical roles played by PCs and MVBs recently demonstrated in tumourigenic processes.

## Introduction

Gastrointestinal stromal tumours are the most common mesenchymal (non-epithelial) neoplasms of the human gastrointestinal tract. GIST was historically classified either as a smooth muscle tumour or a nerve tumour, the other mesenchymal neoplasms of the GI tract. In 1984 Mazur and Clark 1 studied a significant number of spindle cell tumours of the GI tract and found no definite clue to their histogenesis either by electron microscopy or immunohistochemistry. They proposed the more generic designation ‘gastrointestinal stromal tumour’ to help distinguish this type of tumour from true smooth or Schwann cell neoplasm. GIST has been considered to be a particular tumour type since the revelation concerning the gain of function mutations of c-Kit in GISTs by Hirota *et al*. 2.

Typically, GISTs express the c-Kit receptor tyrosine kinase (KIT), and they are thought to derive from ICCs or an ICC progenitor through somatic mutation, based on immunophenotypical and ultrastructural similarities 3–5. Moreover, a mouse model for the study of the constitutive activation of Kit in oncogenesis has been produced demonstrating that constitutive Kit signalling is critical and sufficient for the induction of GISTs (indistinguishable from human GISTs) and hyperplasia of ICC 6.

Recently, DOG1 (=ANO1) antibody has been reported as a highly sensitive and specific marker for GISTs. It is expressed ubiquitously in GISTs irrespective of Kit or PDGFRA 7. ANO1 regulates proliferation at the G1/S transition of the cell and may play a role in tumourigenesis 8.

There have been several reported series of ultrastructural studies of GISTs documenting ultrastructural variability in tumour cells. Attempts have been made to identify ultrastructural features of ICCs within GISTs with considerations about their histogenesis, with contradictory conclusions 3, 9–11. Most of the reported series of ultrastructural studies dealing with GISTs have focused on the identification of neural and smooth muscle features consistent with their origin from a multipotential precursor cell. In contrast to small intestine and rectal GISTs, gastric GISTs constantly exhibit features of myoid differentiation. We have previously reported the presence of a primary cilium in ICCs in the rabbit and rat duodenum 12, 13, as well as in ICCs from human stomach (observations not published). In addition to the ultrastructural features of GISTs described to date, we examined four cases of gastric GISTs looking for the primary cilium to provide additional ultrastructural data to support the currently prevailing concept that GISTs derive from ICCs.

In the last decade, beginning with important electron microscopic studies and culminating in immunolocalization combined with molecular technology much has been learned about PC in mammals and their functional importance has been established. Cilia are membrane-bounded, centriole-derived, microtubule-containing projections from the cell surface. Single non-motile 9+0 cilia are now called PC and form the basis for various specialized sensory structures that are critical for cell signalling 14, 15. In vertebrates, the primary cilium acts as a hedgehog signalling centre. The relation between PC and proliferation and differentiation during development, has led to the investigation of the role of PC in cancer. Hedgehog signalling can promote cell proliferation, and excessive hedgehog signalling can lead to cancer 16. The presence of primary cilium in GISTs has not been previously described.

## Materials and methods

### Materials

Four cases of stomach tumours that fulfilled the histological criteria of GISTs were examined in the study. The tumours were composed of spindle and epithelioid cells arising in the wall of the stomach. They were retrieved from the files of the Department of Pathology at the Zaragoza University Clinic Hospital. Immunohistochemical studies using the EnVision Dako Autostainer System were performed to confirm their diagnosis by using CD117 and CD34 (expressing intense uniform positivity), smooth muscle actin (inconstantly focally positive) and desmin and S100 protein (negative). These results have been summarized in Table 1.

**Table 1:** Immunohistochemical profile of tumours included in the study

	CD 117, c-kit	CD 34	SMA	DESMIN	S100
Case 1	+	+	−	−	−
Case 2	+	+	+/−	−	−
Case 3	+	+	+/−	−	−
Case 4	+	+	−	−	−

+: A strong cytoplasmic staining is observed in most of the tumour cells.

−: No staining is observed or staining present in isolated tumour cells.

+/−: The immunostaining pattern is heterogeneous and positively stained tumour cells are focally distributed.

### Transmission electron microscopy (TEM)

After the tumour extraction, samples (about 1–1.5 mm^3^) were washed in phosphate buffer and fixed with 2.5% glutaraldehyde and 2% paraformaldehyde overnight at room temperature, washed in 0.1 M phosphate buffer for 5 min., post-fixed with 2% osmium, rinsed, dehydrated in graded acetones (30%, 50%, 70% with 2% uranyl acetate, 90%, 100%), cleared in propylene oxide and embedded in araldite (Durcupan, Fluka AG, Buchs SG, Switzerland).

Semi-thin sections (1.5 μm) were cut with a diamond knife, stained lightly with 1% toluidine blue and examined by light microscopy (Olympus BX51 microscope, Olympus Imaging Corporation, Tokyo, Japan). Later, ultrathin (0.05 μm) sections were cut with a diamond knife, collected on Formvar coated single-slot grids, counterstained with 1% uranyl acetate and with Reynold's lead citrate for 10 min. and examined under a FEI Tecnai G2 Spirit TEM. The images were captured with Advanced Microscopy Techniques, using a Corp. Charge-Coupled Device (CCD from Danvers, MA, USA) imaging system.

## Results

**Fig. 1:**
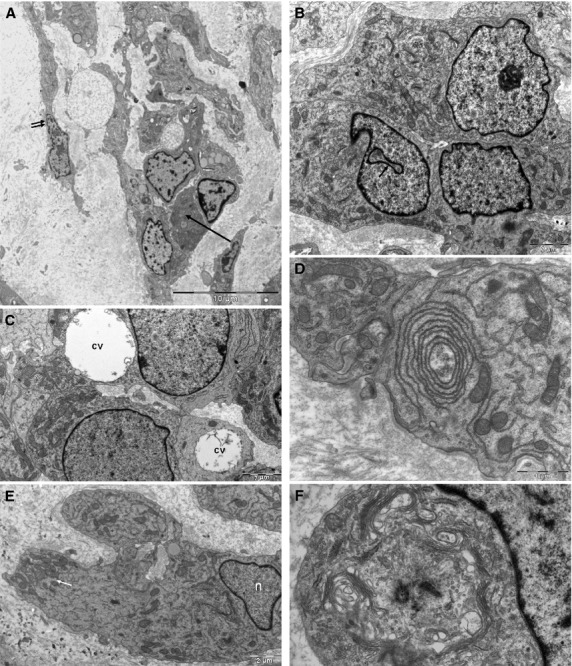
Gastric GISTs (**A**). Heterogeneous aspect presenting cells, which may range from polygonal epithelioid to spindle. Interconnected epithelioid cells may form small cellular groups (arrow). Spindle cell (double arrow). (**B**) A small group of epithelioid cells: nuclei with finely granular chromatin and prominent nucleoli. Some cells present characteristic deep invaginations of the nuclear envelope (arrow). (**C**) Epithelioid cell shows large paranuclear cytoplasmic vacuoles (cv). (**D**) Cytoplasmic organelles. Stress on the concentric arrangement of narrow cisternal lumen of endoplasmic reticulum. (**E**) Accumulation of Mitochondria (arrow) on the edge of an epithelioid cell (n: nucleus). (**F**) Centrosome close to the nucleus, surrounded by multiple Golgi dictyosomes.

The available material for ultrastructural examination of the gastric GISTs revealed a notable variability in tumour cells. The tumours presented a heterogeneous aspect, predominately epithelioid; in minor proportions, a variable presence of phenotypically different spindle cells was found (Fig. 1A). Discohesive oval to polygonal cells separated by a loose stroma were present in every case.

The epithelioid cells always presented plump polygonal cytoplasm containing slightly displaced round nuclei with a finely distributed euchromatin and slightly undulating envelope. The inner nuclear membrane showed a thin frame of marginal heterochromatin. Nucleoli were prominent. The nuclei occasionally exhibited some deep invagination (Fig. 1B). In one cases, some of the cells contained paranuclear membrane-bound cytoplasmic vacuoles (Fig. 1C).

The cytoplasm contained abundant rough and smooth endoplasmic reticulum, which sometimes appeared in a concentric arrangement (Fig. 1D) and numerous large mitochondria scattered throughout the cytoplasm and occasionally grouped into one end of the cell (Fig. 1E). The Golgi apparatus was well developed and in some cells surrounded the centrosome, which was composed of two centrioles and pericentriolar material (Fig. 1F).

**Fig. 2:**
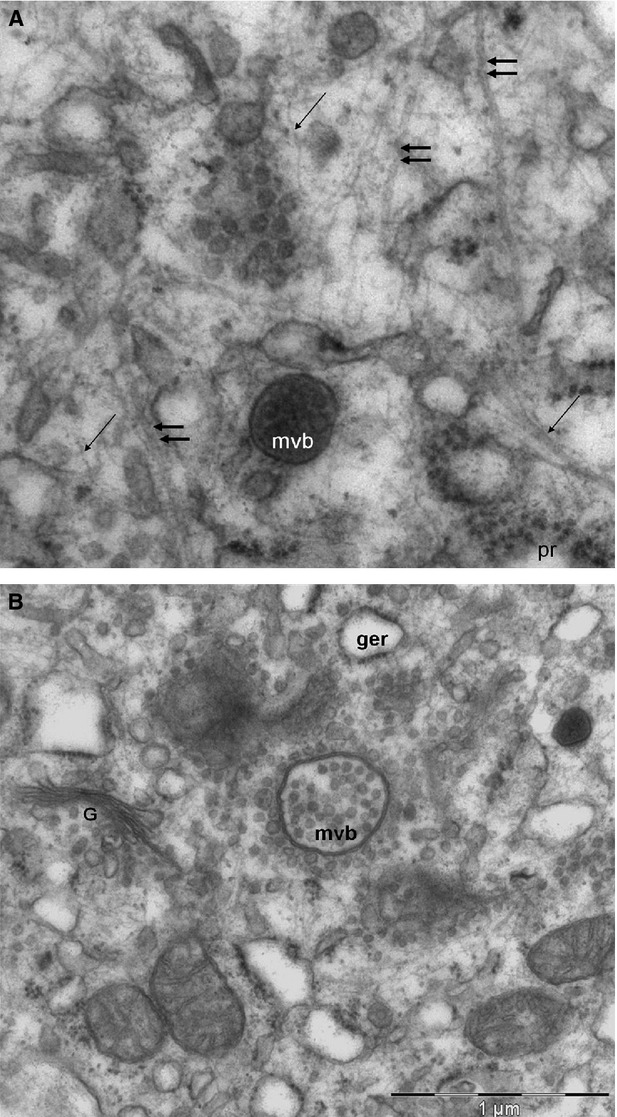
Gastric tumoural cell (**A**). In the cytoplasm can be seen isolated microtubules (double arrow), abundant filaments (arrow), and free polyribosome (pr). (**B**) Multivesicular body next to a Golgi dictyosome, surrounded by small vesicles.

**Fig. 3:**
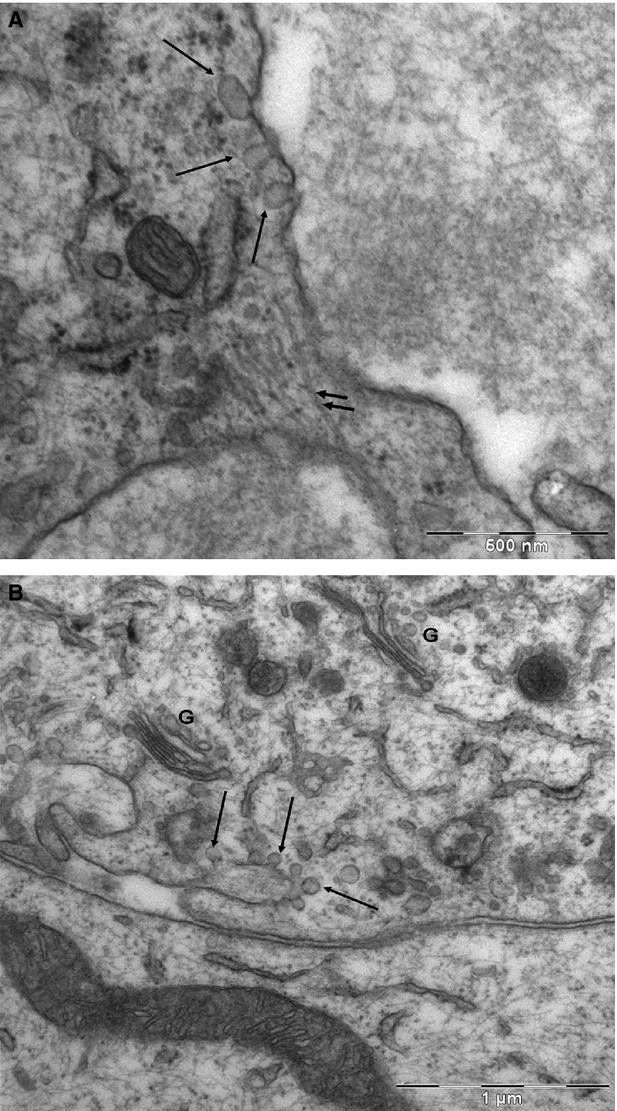
Gastric tumoural cell (**A**). Pinocytotic vesicles on the surface of the plasma membrane. Microtubule (double arrow). (**B**) Pinocytotic vesicles in an invagination of the plasma membrane (arrow). G: Golgi apparatus.

Other ultrastructural features of the neoplastic cells included prominent cytoplasmic filaments, which frequently formed aggregates, isolated microtubules, free polyribosomes and multivesicular bodies (Fig. 2A). The multivesicular bodies were sometimes observed surrounded by small vesicles (Fig. 2B). Tumour cells having dense-core membrane-bound granules suggestive of neuroendocrine differentiation were not revealed, but endocytosis or exocytosis was demonstrated by the presence of pinocytic vesicles on the surface (Fig. 3A) or in small invaginations of the plasma membrane (Fig. 3B).

**Fig. 4:**
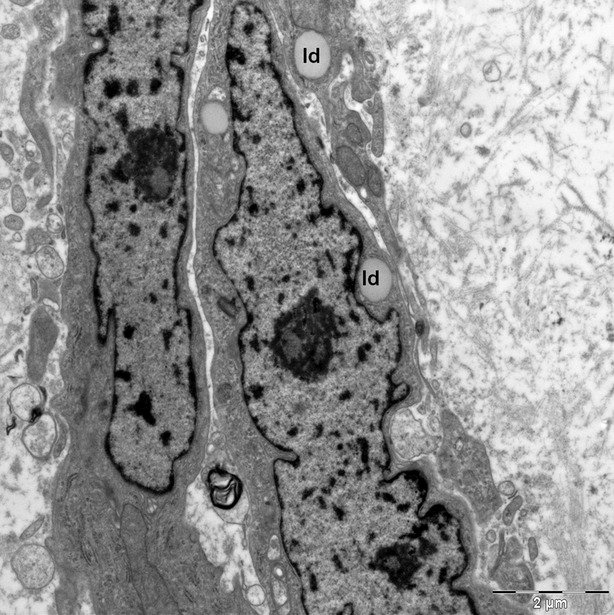
Gastric tumoural spindle cells show chromatin compacted into small clumps and prominent nucleoli. Lipid droplets can be seen in the cytoplasm (ld).

The spindle cells had the same features, but the nucleus showed chromatin compacted into small clumps and prominent nucleoli (Fig. 4).

**Fig. 5:**
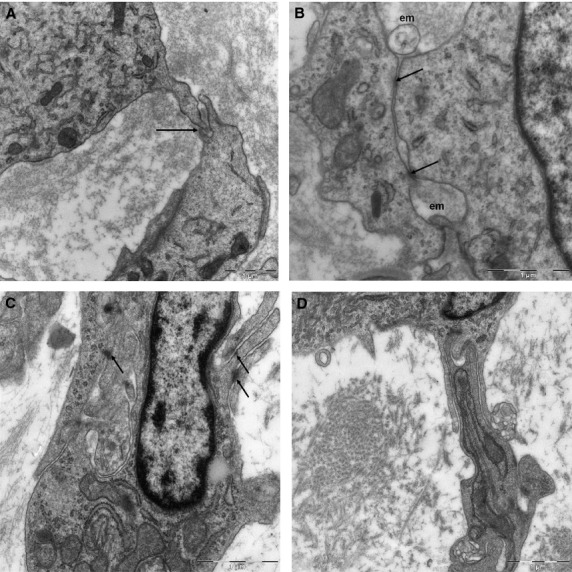
Gastric tumoural cells (**A**). Peg and socket junction between two tumoural cells (arrow). (**B**) Intercellular gap-like junctions (arrow) and expanded spaces occupied by the extracellular matrix are observed between both cells (em). (**C**) Desmosome-like junctions (arrow) between spindle cell prolongations. (**D**) Interdigitating filopodia-like cytoplasmic cell processes.

A fairly constant feature seen in both the spindle and epithelioid tumour cells was the presence of interdigitating cell processes and invaginating cellular protrusions (peg and socket junctions) between cells (Fig. 5A). There were frequent symmetric membrane thickenings consistent with close contacts (intercellular gap-like junctions; Fig. 5B) between neighbouring cells and numerous desmosome-like (Fig. 5C) junctions on interdigitating filopodia-like cytoplasmic cell processes (Fig. 5D). The extracellular matrix was composed of loose flocculent or fibrilar material with scattered banded collagen fibres (Fig. 5A–D). Very exceptionally, obvious external lamina was noted. Skeinoid fibres were not identified.

**Fig. 6:**
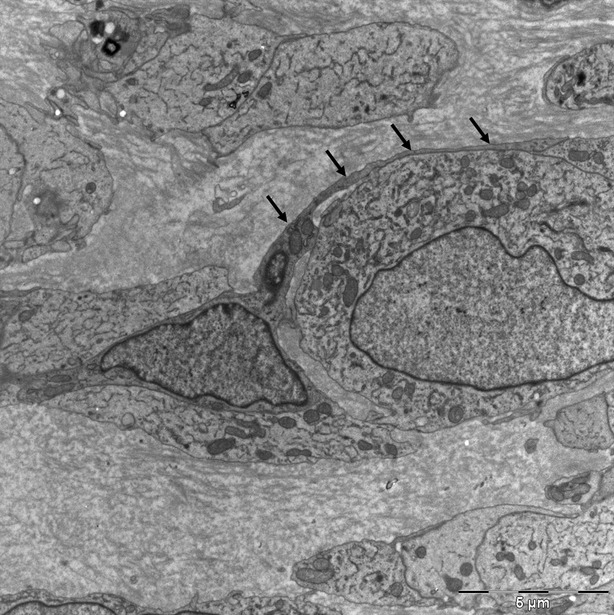
Gastric tumoural cells. Some cells show ICC features such as scarce cytoplasm and long-fine cytoplasmic extensions (arrow) connecting with neighbour cells.

Only a few cells displayed the phenotypic characteristics of the ICCs and these were reminiscent of adult ICCs. They showed a voluminous nucleus surrounded by a narrow perinuclear cytoplasm, which expanded with long slender cytoplasmic processes. These prolongations established close contacts with other cells (Fig. 6).

**Fig. 7:**
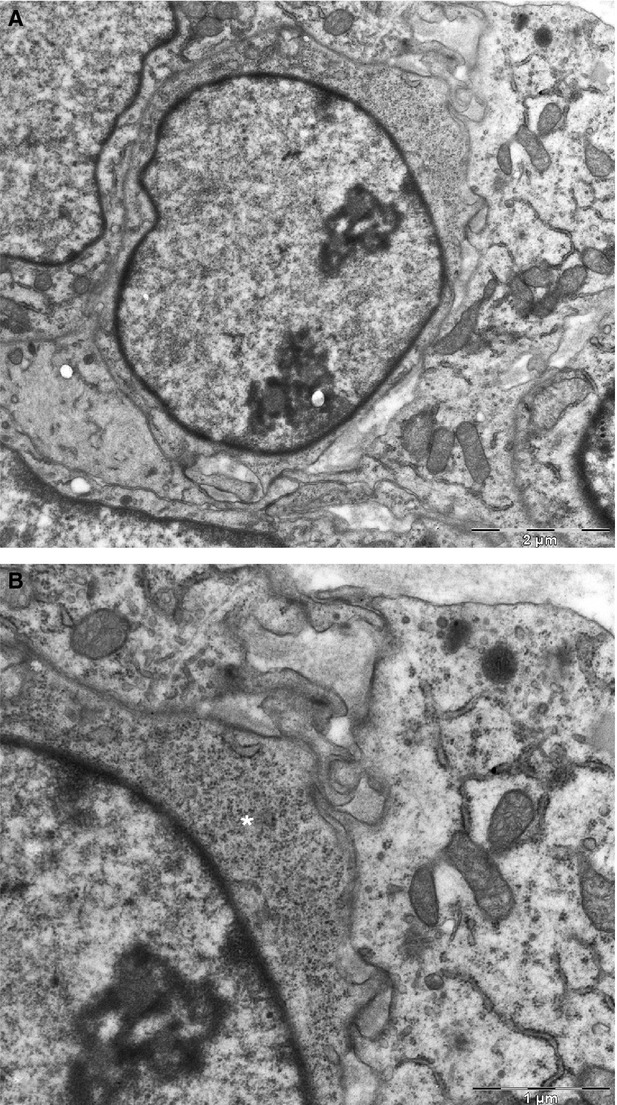
A round gastric tumoural cell presenting undifferentiated characteristics (**A**). A large nucleus-cytoplasm ratio. (**B**) Cytoplasm contains numerous free polyribosomes (asterisk). Magnification of 12A.

Another type of tumour cell observed was a small round cell, which had a rather larger nucleus-cytoplasm ratio with simpler cell body and fewer cytoplasmic organelles, containing numerous free polyribosomes (Fig. 7A and B). Nucleolus was prominent. The overall appearance of these cells was suggestive of undifferentiated cells.

**Fig. 8:**
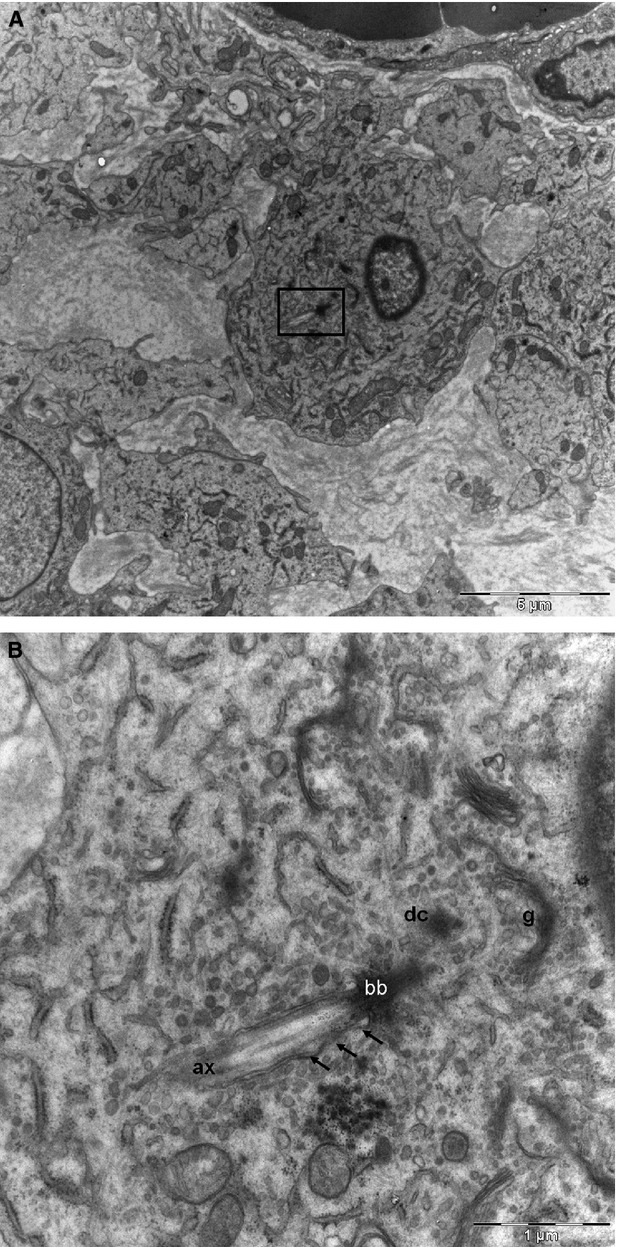
Epithelioid tumoural cell (**A**). PC emerges from the cytoplasm to the cell surface (insert). (**B**) Ultrastructural charcetristics of PC: basal body (bb), axoneme (ax) and ciliary pocket (arrow). Daughter centriole (dc) was close to the Golgi complex (g). Magnification of 8A.

**Fig. 9:**
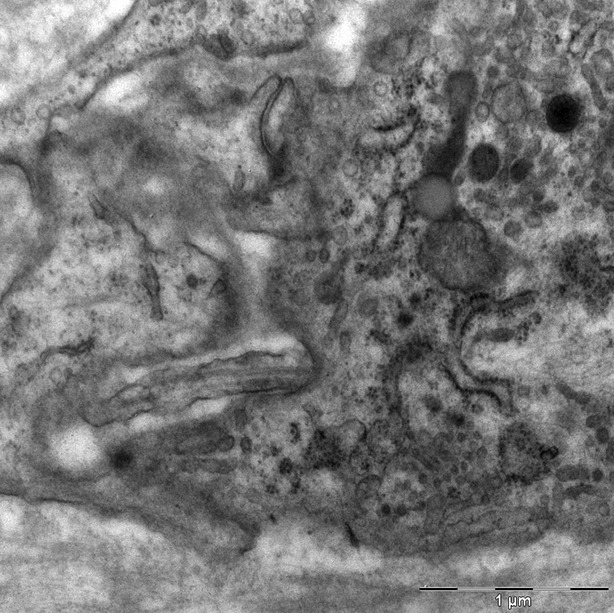
Gastric tumoural epitheloid cell. Longitudinal section of PC projecting into the extracellular space. It has a length of 1–2μ.

**Fig. 10:**
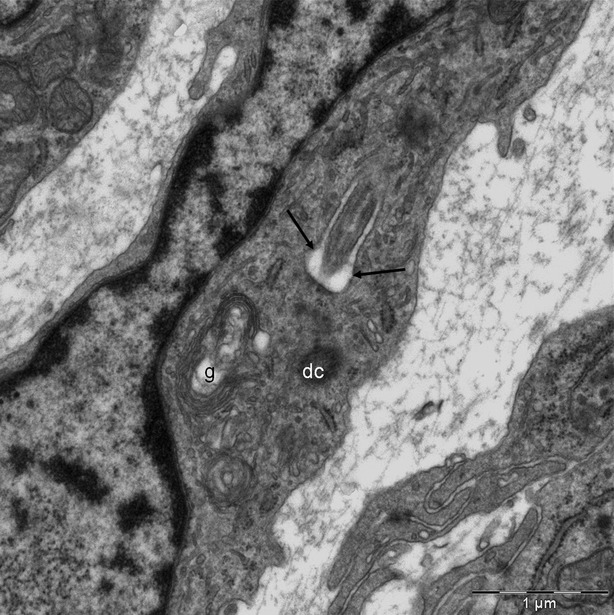
Tumoural spindle cell. The proximal region of the cilium is located within an invagination of the plasma membrane: the ciliary pocket (arrow). Daughter centriole (dc). Golgi complex (g).

**Fig. 11:**
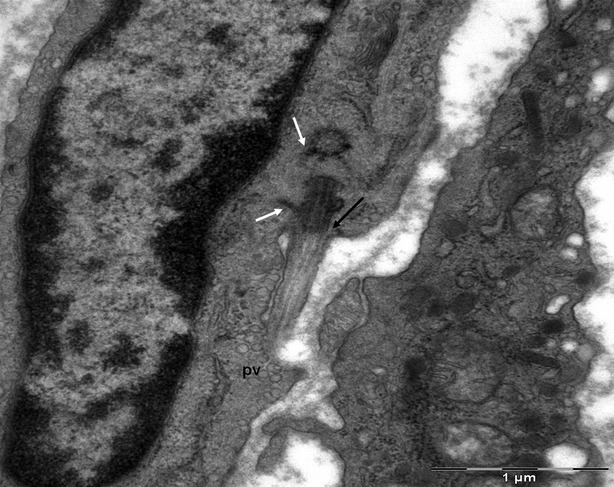
Tumoural spindle cell showing diplosome: the PC emerges from a typical basal body. Both centrioles present radial satellites (white arrow). Alar sheets (arrow). Pinocytotic vesicles (pv) are concentrated next to ciliary membrane.

The most interesting ultrastructural finding in the tumour cells was the presence of PC. This was present in all gastric GISTs, though it was almost inappreciable and was found only after a diligent search (slightly higher than 5%). The PC derived from the parental centriole of the diplosomal pair emerged close to nucleus (Fig. 8A). The axoneme was a direct extension of a typical basal body and contained nine outer doublet microtubules. The daughter centriole was close to the Golgi complex (Fig. 8B). The PC exhibited a 9+0 pattern because the central pair of microtubules was not observed in any of the serial sections and it lacked motility-related components such as inner and outer dynein arms and radial spokes. It was projected into the extracellular space and had a length of about 1–2 μm (Fig. 9). Some TEM images indicated that the cilium remained partially intracellular within a membrane invagination, the ciliary pocket (Fig. 10). Both the ciliary pocket and the axonema were surrounded by a membrane that was continuous with the plasma membrane of the cell. This ciliary pocket appeared as an endocytic domain for endocytosis by vesicles formation (Fig. 11). In this same figure, we can see the basal body from which distal end transitional fibres (alar sheets) extended outwards from each microtubular triplet and formed contacts with the plasma membrane. This point of contact defined the boundary between the plasma membrane and the ciliary membrane. The parental and daughter centrioles displayed peripheral satellites. The axoneme of the cilium lay parallel to the cellular membrane, where multiple pinocytotic vesicles appeared.

**Fig. 12:**
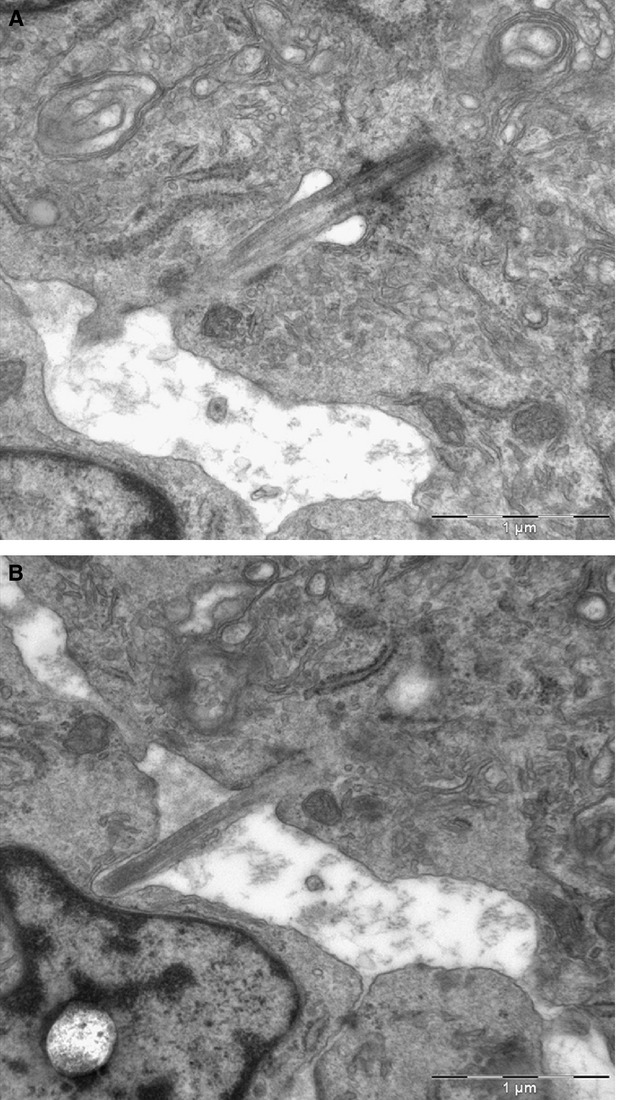
Ultrathin serial sections of a tumoural epitheloid cell (four sections not showed in Figure exist between A and B). (**A**) Intracellular portion of the ciliar axonema lodged in the ciliary pocket. (**B**) The extracellular portion of the axonema protrudes outside the membrane of the next cell.

The serial ultrathin micrograph study let observe the intracellular disposition of the axonema lying deep in the ciliary pocket (Fig. 12A) as well as protruding outside the cell even putting out the shape of the next cellular membrane (Fig. 12B).

## Discussion

Gastrointestinal stromal tumours have been recognized as a biologically distinctive tumour type, different from smooth muscle and neural tumours. Most GISTs are positive for KIT (CD117) and 70–80% of GISTs are positive for CD34 17–19. The c-Kit positivity of GISTs parallels that seen in ICC. Based on this, and on the expression of other markers such as CD34 and an embryonic form of smooth muscle myosin heavy chain in GISTs and ICCs 20 the origin from ICC, or from a precursor of these cells, has been proposed.

To shed further light on the histogenesis of GISTs, attempts have been made to identify ultrastructural features of ICCs within GISTs.

Our study, in accordance with some authors 3, 9, 11, 21, indicates that all gastric GISTs contain cells that exhibit some ultrastructural characteristics of ICCs 22, 23 including actin-like filament bundles, numerous large mitochondria, abundant smooth endoplasmic reticulum, microtubules, caveolae, interdigitating cytoplasmic processes, incomplete external lamina and intercellular gap-like junctions.

Our own observations of rat and rabbit intestinal specimens 12, 13 and the findings of Min in humans 11 have shown that ICCs around myenteric plexuses and in submucosa have a close relation with nerves, vessels and muscular cells, which perhaps constitute the most reliable ultrastructural feature for their identification under an electron microscope. This close relationship has important roles in gastrointestinal motility and transmission of nerve signal from nerve cells to smooth muscle cells in normal tissue. In this study, tumour cells in gastric GISTs, independent of their morphology, do not appear to have the close association with nervous trunks and the close relationship with blood capillaries and muscular cells seen in normal ICCs. This fact would involve the loss of the normal functionality in tumoural cells. Robinson *et al*. 10 have indicated that the unique combination of several distinct ultrastructural features that identify ICCs are not present in GISTs, suggesting that although the tumour may have originated from ICCs, the tumour cells have differentiated away from the original ICC phenotype. Nevertheless, as we have shown in our results, some cells not only have features seen in normal ICCs, but they can also be positively identified as being adult ICC-like. Other studies have analysed ultrastructructural characteristics of GISTs arising from various regions of the GI tract and established considerable significant regional differences according to the site of their origin as well as certain similarities 9, 11. This has led some authors to support the concept that GISTs recapitulate the phenotype of ICCs 9 and others to propose the origin of GISTs in another cell, a common progenitor cell, which should have the potential to differentiate towards the cells described in GISTs 11. Gastric GISTs show features to myoid differentiation also to ICCs. These findings argued for the origin of gastric GISTs, suggesting that it may reside in primitive mesenchymal cells, which are able to differentiate to smooth muscle cell as well as to ICCs.

Nevertheless, in our opinion, the histogenesis of GISTs should not depend on ultrastructural morphology alone or on similarities between tumour cells and potential progenitor cells, given the plasticity of both ICCs 23 and stem tumour cells 11. Furthermore, the presence of some cytoplasmic features such as networks of intermediate filaments or bundles of thin filaments and microtubules, or the presence of surface pinocytic vesicles along the cytoplasmic membrane that have been used to argue in favour of a histogenetic origin are not in fact indicative of such an origin, but are perhaps indicative of a specific line of differentiation or a common machinery of the cell.

The presence of a PC in ICCs has been reported by us in rabbit and rat duodenum 12, 13 and by other authors in the mouse stomach 24 and in ICC-like cells in rat mesentery 25. In a similar way, the PC is a constant feature in the ICCs from the human stomach myenteric plexus (observations not published).

In addition to the ultrastructural features of GISTs described so far, we report for the first time, as far as we know, the existence of the PC, an organelle present in each of cases of gastric GISTs that we have studied. Our results demonstrate that PC were present in epithelioid and spindle cells, but were almost inappreciable under ultrastructural observation that they were found only after a diligent search. However, only some tumoural cells (not entire GISTs) present PC. Its presence seems to be important for the maintenance of the differentiated stage of the cell. Meanwhile, PC necessarily disappears in mitosis as basal bodies would migrate to form the mitotic spindle. As consequence, the role of the loss of PC in GIST cells may be critical for proliferation or cytogenesis.

We also report the presence of exosomes and MVBs in gastric GISTs that have been referred to in a recent report in ICCs like cells (‘telocytes’) in GISTs 26.

Both organelles were discovered and described many years ago, but curiously neither has attracted particular interest until recently. In the last decade, internalized signalling molecules and their receptors have been found to accumulate in these organelles and have been implicated in cell-to-cell communication. Recent studies show that both PC and MVBs play critical roles in tumourigenesis 27–30.

The PC is a dynamic organelle whose cyclic formation and disassembly is inherently related to the cell cycle. New data provide evidence that ciliary resorption not only precedes but also influences cell cycle re-entry 14. It is also known that aberrant activation or the absence of ciliary hedgehog signalling is correlated with uncontrolled cell division, oncogenesis and cancer 27–29. Moreover, several mouse models of Hh pathway activation may be linked to pancreatic ductal carcinoma 18, skin basal cell carcinoma 27, meduloblastoma 28, 29 and rabdomiosarcoma. Sonic Hh overexpression plays a crucial role in human *Helicobactor pylori* induced carcinogenesis 31.

Although cilia have been reported to be absent in most cancers to date, an increasing number of tumours harbouring PC are now being found.

The prevalence of cilia on human tumours remains unclear, and their role of cilia in cancer is just beginning to be explored. PC have been found by immunofluorescence method to be decreased in cells of several types of cancer, including cancers of the kidney, skin, brain, pancreas, breast and prostate, compared with their normal cellular counterparts 32–37.

Aberrations in ciliogenesis and the resultant absence of mature PC in cultured astrocytoma/glioblastoma cells have been described 35. This study does not demonstrate ciliogenesis defects and the PC appear structurally normal.

Primary cilia have been reported to have a dual opposing function in the development of brain tumours in mouse models of cancer. Thus, PCs are either required for or inhibit medulloblastoma formation, depending on the initiating oncogenic event 29. These seemingly paradoxical effects are also described in skin basal cell carcinoma 27.

In a study of the fate of this organelle during cancer development, Kim *et al*. 32 found that PC assembly is actively suppressed by excessive K-ras signalling in pancreatic ductal adenocarcinoma cells and malignant melanoma cells.

On the other hand, our results show the association of the PC with endocytotic vesicles which may be crucial to cell signalling. This finding corroborates evidence given in a recent study 35. Exosomes are small membrane vesicles of endocytic origin formed in endosomal compartments called multivesicular bodies that package and store signalling molecules and are thought to play important roles in intercellular communications. A large amount of recent data highlight their possible functions as messengers during the development of tumours 38, 39. Although several groups defend the hypothesis that tumours secrete exosomes to promote their growth by inhibiting antitumour immune responses, or by promoting angiogenesis or migration outside the tumour bed to form metastases, this has not been proved. It is worth noting that mRNA has also been described in microvesicles (or mixed exosome/microvesicle preparations) released by tumours or embryonic stem cells 40.

To the best of our knowledge, the presence of PC in GISTs has not been previously reported. This study shows that the presence of PC and MVBs in gastric GISTs parallels that seen in ICC. This phenomenon may or may not be of significant histogenetic value. More importantly from a biological standpoint, this study provides new ultrastructural data on the mechanisms of cell-to-cell communication in tumours.
